# Assessing the Awareness of Agents Involved in Issuance of Death Certificates About Death Registration Rules in Iran

**DOI:** 10.5539/gjhs.v7n5p371

**Published:** 2015-07-07

**Authors:** Abdollah Mahdavi, Shahram Sedghi, Farahnaz Sadoghi, Farbod Ebadi Fard Azar

**Affiliations:** 1 Health Information Management Department, School of Health Management and Information Sciences, Iran University of Medical Sciences, Tehran, Iran; 2 Medical Librarianship and Information Sciences Department, School of Health Management and Information Sciences, Iran University of Medical Sciences, Tehran, Iran; 3 Public Health Department, School of Health, Iran University of Medical Sciences, Tehran, Iran

**Keywords:** death registration system, death certificate, mortality coding, awareness, performance, ICD-10

## Abstract

**Introduction:**

In the death registration system, issuance of death certificate, as a binding rule, is considered among the major necessities of preparation of death statistics. In order to prepare death statistics that are adequately valid for subsequent applications, it is necessary to properly encode death certificates and fully follow rules on causes underlying death. This study aimed to assess the awareness and performance of agents involved in issuance of death certificate in the national death records system.

**Methods:**

It was a descriptive cross-sectional research, which was performed from September 2013 to March 2014 on 96 agents involved in issuance of death certificate Imam Khomeini, Alavi, Fatemi and BuAli education and treatment centers of Ardebil University of Medical Sciences. The population included faculty staff physicians, residents and health information management staffs. The research scale was also a researcher-made questionnaire that questioned the demographic information as well as awareness and performance of participants regarding death certificate coding rules. Research data was analyzed based on descriptive statistics and the chi-square test method in the SPSS software at a confidence level of 95%.

**Findings:**

A total of 34.42% of participants were aware of the general rules on issuance of death certificates while faculty staff higher specialists (41.67%) and clinical coders (38.34%) with five years of experience demonstrated the highest awareness levels. Only 23 participants (24.6%) were trained to issue death certificates. A total of 76 participants (79.3%) announced their need for learning how to complete death certificate forms on a constant basis. The awareness of participants about the general principle was assessed to be low (30.25%). Moreover, their awareness of selection rules and modification rules was low (27.75%) and moderate (45.25%), respectively. The chi-square test revealed a significant relationship between work experience and awareness of participants about coding rules (P=0.001), but no significant relationship was observed between education and awareness of coding rules (P=0.497).

**Conclusion:**

The awareness of participants about rules on coding death causes and their performance in this field was so satisfactory. That is to say, the awareness of faculty staff and health information management staffs was unexpectedly low. Seemingly, lack of adequate training is an international issue that causes mistakes in the recording of information on mortality. Hence, a short-term solution is to train faculty staff and residents and also revise the training provided to health information management staffs. As a long-term solution it is possible to provide related courses to general practitioner students.

## 1. Introduction

The most important source of information used by health organizations to obtain information on the population is vital statistics. Vital statistics include information obtained from regular, careful and continuous records of vital events and the most important vital event is death (Khosravi, Taylor, & Lopez, 2007). Mortality information and identification of the causes of death are important means of monitoring and assessing the level of health of a society. These measures also play an important role in prioritizing health issues. Hence, today, rules and systems for collection and registration of death statistics are developed with a special attention that is paid to the registration of death events and their causes (Mohammad, 2005). Careful drawing of death and illness is the major way of increasing the lifetime and health of members of a society. This picture helps identify and confront the causes of death (Health Deputy, UMUS, n.d.). Mortality information used for monitoring the health of the population, allocating budgets, adjustment research targets and estimating the total health of the population (Goldacre & Griffith, 2003; Ly, Xing, Klevens, Jiles, & Holmberg, 2014). Accordingly, valid information on death causes and variations of this information form one of the significant bases of health planning, management and evaluation in every country. If death statistics are prepared with more detail, care and credibility, it is easier to make more systematic decisions and supervise the society more effectively (Zanjani, Shadpure, Mahriyar, & Mohammad, 2000). Information on death causes has been used for years as the means of monitoring overall health improvement and prioritizing health issues and measures (Greenberg, 2001). By using health indicators (especially mortality indicators) it is possible to best determine health and care priorities (Rosenberg, 1999).

As mentioned, the death statistics obtained from the vital events registration system is an important means of proper management and organization of the society at all levels (Zanjani, Shadpure, Mahriyar, & Mohammad, 2000). These statistics are also the basis of data on death certificates issued by physicians as those who are responsible for treatment of patients. Hence, failures in completion of death certificates undermine the accuracy of health programs and proper administration (Lua, Leeb, & Chou, 2000). Therefore, more detailed and systematic death certificates, facilitate coding and increase the precision of results (Reza, 2005). As a result of this improvement, decisions will be made with more awareness and wisdom. However, usually when death statistics are precise and accurate in a country, death causes are identified and determined with more errors! (Schadé, 1987).

Death statistics published by different centers and countries vary to a great extent. In order to produce death statistics that are considered valid at international level it is necessary to use uniform definitions and formats (Lu, Hsu, Bjorkenstam, & Anderson, 2006). Hence, the World Health Organization (WHO) presents a new global standard for death certificates in the second volume of the tenth edition of book International classification of Diseases (ICD-10). This organization has also issued instructions on completion of death certifications, selection of a death cause and encode of certificates. Therefore, ICD defines a process for preparing and comparing death statistics in the form of internationally area (WHO, 2010).

Accurate register of death causes requires an understanding of the difference between death causes and its mechanisms as well as an understanding of the immediate cause of death and its underlying cause. All of the aforementioned factors are defined in the clinical section of the certificate standard proposed by WHO (Villar, & Pere-mendez, 2007). The underlying cause of death is: a) the disease or injury which initiated the train of morbid events leading directly to death, or (b) the circumstances of the accident or violence which produced the fatal injury. But the immediate and main cause of death, last disease or condition that led directly to the death of the deceased. In addition propose from death mechanisms is not signs and symptoms of (such as heart failure, respiratory) (Mahdipor, 2008).

According to the ICD-10 instructions death causes are encoded based on the death underlying cause (WHO, 2010). It is worth noting that proper determination of the underlying cause of death plays an important role in clinical trials and studies associated with the quality of outputs of the health care system (Gupta, Bharti, Singhi, Kumar, & Thakur, 2014). Although it seems that record of causes underlying death is a simple process, it is associated with several difficulties such as lack of adequate awareness of physicians, their lack of attention to the record of underlying causes, lack of adequate information on the deceased and the complicated process of some illnesses (Moy, Albert, Diener-West, McCaffrey, Scully, & Willson, 2001). According to previous studies, 37% of death certificates are defective in terms of the underlying causes of death. In some case, death certificates are not completed based on reports of death causes (Mackenbach, Kunst, Lautenbach, Bijlsma, & Oei, 2005).

Studies that were performed in United Kingdom indicated that 46% of physicians are not properly aware of instructions on issuance of death certificates (Maudsley & Williams, 2003). In the United States, some of the main causes of errors in death certificates include time restrictions, exhaustion, a lack of awareness of rules on determination of death causes, lack of experience of human forces, and lack of awareness of associated agents about the history of diseases of the deceased (Pritt, Hardin, Richmond, & Shapiro, 2005).

On the other hand, patients’ information can be only useful when it is properly classified and organized through accurate coding (Bancroft & Lee, 2014). Coding is directly associated with data quality. Health information management staffs encode medical records and death certificates in order to provide for recovery of information of patients and injuries (Shorbaji, 2001). They deal with death causes, especially underlying causes, and information recorded in death certificates. In other words, the more the accuracy of the information, the higher is the quality of coding and the easier is interpretation of the associated rules by the encoder (Franca, Rao, & Lopez, 2008; Anderson, 2003). Moreover, the effects of training, experience, characteristics and personal interests of encoders on the reliability of the codes have been proved too (Schelhase & Weber, 2007).

Although numerous studies have been conducted in Iran on the coding of diseases (Farzandipour, 2009; Jahanbakhsh, n.d.), very few studies have been conducted on the coding of death causes (Shokrizadearani, n.d.). Proper completion of death certificate forms and subsequent coding is important while researchers repeatedly observe deficits due to their position and professional communication or are informed of such deficits through their students. Therefore, if these deficits remain unsolved, not only scientific decision making based on professional information is disrupted but also the requirements of patients, dependents, and natural and legal clients are not satisfied as well. Consequently, this leads to a long-term disruption of management of heath care complexes. Hence, the present research aimed to first assess the awareness and performance of faculty staff clinical specialists, specialty residents, and health information management staffs as agents involved in issuance of death certificates in the death registration system. It also aimed to clarify the weaknesses and strengths of the current status and provide necessary and special information/consultations to managers on the selection of useful and effective solutions.

## 2. Materials and Methods

It was a descriptive cross-sectional study that was performed from September 2013 to March 2014. It was carried out as the national trial program for death registration in Imam Khomeini, Alavi, Fatemi and BuAli education and treatment centers that are affiliated with the Ardebil Medical Sciences University. In order to assess the awareness and performance of all of the agents involved in issuance of death certificates, all coding experts (8 persons), clinical department faculty staff (69), and senior residency students (19) except for the students in the radiology, anesthesiology and pathology departments were selected. A total of 96 individuals, except for the aforementioned three groups that are not involved in issuance of death certificates were selected for the research.

Information was obtained through a questionnaire to determine the awareness and performance of faculty staff, residents, and health information management staffs. The questions included in the questionnaire were formulated based on the rules and guidelines defined for determination of death causes in the WHO death certificate standard, the second volume of ICD-10, the Iranian death certificate form, and all associated circulate.

It included three sections: the first section focused on demographic information and the second section included twenty questions, which were formulated and arranged based on the diagnoses included in the medical record of a deceased patient. The second part was arranged in the form of mortality record guidelines. Each question could be answered by two, three or four options (diagnostic answers) and there was a correct answer (option) to every question that determined the underlying cause of death. The score determined for the correct answer was ten and when no correct answer was provided to a question the score was zero. In order to compare the results of the tests with the results of other studies, the results were expressed in percent. The third part of the questionnaire addressed data necessary for registration of death certificates. Such data included general information and clinical causes of death.

Awareness was rated according to the following classifications: very low (0-20), low (20-40), moderate (40-60), high (60-80) and very high (80-100). The validity of the questionnaire was examined and confirmed by comparing the statements with contents of the guidelines included in the section on “Rules and guidelines for mortality and morbidity coding” of the second volume of ICD-10. The reliability of the questionnaire was also confirmed using the “test/re-test” method. In addition, the Pearson’s correlation coefficient obtained for the questions included in the questionnaire was *r* = 0.94.

Of the 107 questionnaires distributed among the participants, 96 were returned and analyzed. Other questionnaires were excluded from the analysis due to overall deficits, lack of return and selection of more than one option by the respondents. Research data was documented using the SPSS ver.16 software and was analyzed using descriptive data and the chi-square test at a confidence level of 95%.

## 3. Findings

In the research on the status of death registers and awareness also performance of agents involved in issuance of death certificates, 107 questionnaires were distributed and 96 (89.7%) were completed and analyzed. Research findings revealed that 57 participants (59.4%) were male and 39(40.6%) were female. Concerning the education and employment status of the participants it shall be said that 69(71.87%) were clinical department faculty staff (Specialist and higher), 19(19.79%) were clinical residents (students), and 8(8.34%) were health information management staffs (bachelors) with five years of work experience. The clinical majors under study included internal diseases, cardiovascular diseases, nephrology, urology, obstetrics and gynecology, and general surgery. Table (1) shows the information on the participants.

**Table 1:** Characteristics of participants (N =96)

Characteristic		Frequency (%)
**Gender**	female	39(40/60%)
	male	57(59/40%)
**Age(years)**	≥35	12(12/50%)
	36-45	69(71/87%)
	≤46	15(15/62%)
	Average age 36 years/Range 25-56 years	
**Position of educational participants**	Higher Specialist academic staff	5(5/20%)
	Specialist academic staff	64(66/67%)
	Clinical residents (Students)	19(19/79%)
	Health information management staffs	8(8/34%)
**Time working (years)**	≥5	29(30/20%)
	>5	67(69/80%)

The average awareness of the research population about all rules was 34.42% (low). Moreover, faculty staff (higher than clinical specialist) and health information management staffs with more than 5 years of professional experience demonstrated the higher level of awareness (41.67 and 38.34%). Only 23 participants (24.6%) had been trained on issuance of death certificates in medical sciences university of Iran. a total of 76 participants (79.3%) announced their need for training on completion of death certificate forms. However, 60 participants (62.5%) reported on their awareness of filling death certificate forms while 37 (38.5%) reported that they had never issued such a certificate. Moreover, 89 participants (92.7%) were aware that the national ID of the deceased is required for completion of death certificates.

**Diagram 1:**
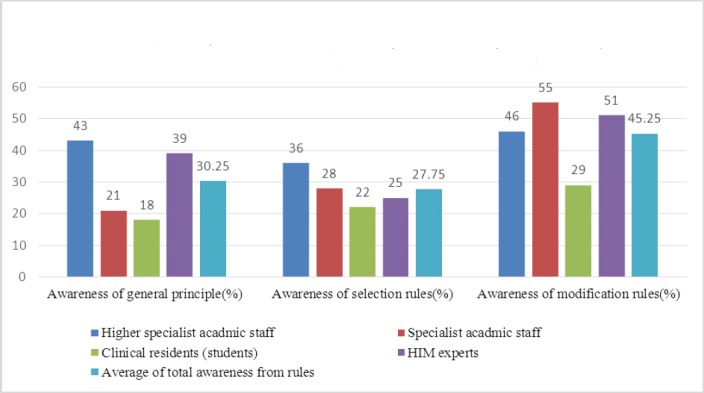
Relative frequencr awareness of agents issuance of death cerificates about cause of death registration rules based on level of educational

The awareness of the participants about general principle, selection and modification mortality registration rules was 30.25%, 27.75% and 45.25%, respectively. The highest level of awareness about modification rules was demonstrated by specialists (55% or moderate) while the lowest level of awareness about general principle was demonstrated by residents (18% or very low) (Diagram 1). The level of awareness of information health management staffs about selection rules was low (25%). Results of the chi-square test showed a significant relationship between work experience and awareness of participants about coding rules (P=0.001), but no significant relationship was observed between education and awareness levels (P=0.497).

The most common problems of physicians with filling death certificate forms were caused by complexity of the death case (52.4%), lack of training (48.2%), and lack of understanding of the terms used in the form (28.9%).

## 4. Discussion

A total of 34.42% of the research population was aware of mortality rules and guidelines. Moreover, specialist faculty staff (41.67%) and health information management staffs (38.34%) with five years of experience demonstrated the highest level of awareness. Only 23 (24.6%) physicians were formerly trained on issuance of death certificate. The awareness of participants of general principle, selection and modification rules on mortality was 30.25% (low), 27.75% (low) and 45.25% (moderate), respectively. The most common problems of physicians with completing death certificate forms were complexity of the case (52.4%), lack of training (48.2%), and lack of understanding of the terminology used in the forms (28.9%).

Result of the research regarding awareness of the agents (clinical department faculty staff, residents and health information management staffs), which was equal to 34.42%, reflected the low level of awareness of the research population. In a study which was performed based on UK mortality record rules it was reported that 54% of the research population was aware of death certificate issuance guidelines and demonstrated a moderate level of awareness (Al-Kubaisi & Horeesh, 2013). This is caused by the positive and responsive attitude of the patients and encoders to identification of death causes. This attitude has improved the quality of death certification documents in UK. Result of their study does not comply with the results of the present research.

In another study (Lakkireddy et al., 2007), participants only demonstrated 19% of precision in issuing and completing death certificates and physicians demonstrated a very low level of awareness of the associated procedures. So, results of this study comply with the results of the present study. To resolve this problem, it is necessary to provide formal training to the agents that are involved in issuance and completion of death certificates. Because one of the most important factors is determining the precision and accuracy of death certificate data, physicians education. The training of physicians and other agents involved in issuance and completion of death certificates are confirmed by most researchers. Fortunately, in this study 79.3% participants announced their need for learning how to complete death certificates on a daily basis. This finding is fully in line with results of the study performed in Kansas City (Lakkireddy, Murray, Basarakodu, & Vacek, 2004). In that research 80.5% of participants also announced their need for more training on this field.

Participants believe that due to the complicated details and multiplicity of rules on determination of underlying, intervening and direct causes of death (especially selection, modification rules and general principle), it is necessary to take regular training courses to remember and review the rules on completion of acceptable death certificates.

A study in Khartoum (El-Nour & Ali, 2007) shows that 94.9% of its participants agree that physicians are responsible for determining the causes of death and completing death certificate forms. This finding is pretty close to the finding of the present research (95.2%). However 39.4 and 27.1% of the participants acknowledge that the head nurse or is responsible for the registration of the death could be related in order to complete the section in the form of a certificate of death sharing would be better. It may be a result of practical experiences and expert opinions of some physicians with the tendency of other health care personnel (such as nurses and head nurses) to take part in completion of death certificate forms.

About 64.8% participants have expressed that they were faced with complicated and problematic details in filling death certificate forms. This result complies with the results of the study (Johansson & Rosenberg, 2006) that was performed to assess methods of measuring the accuracy of death certificates. 42.8% of participants reported a lack of formal training in the course of study and in the beginning of practice as the common cause of such problems. This result is in line with the result of studies performed in United Kingdom and United States (Comstock & Markush, 1986). Research findings suggest that 50% of general practitioners are not adequately trained on issuance of death certificates.

Similarly, a Nigerian study (Izegbu, Shittu, & Akiode, 2006) revealed that 71% of participants were not formally trained on completing death certificate forms. This finding suggest that lack of training is an international issue that causes errors in the record of mortality information. This issue can be a reason for stressing the importance of provision of formal standard trainings on completion of death certificate forms to all physicians and agents in the world. Lack of proper training may bring about difficulties in understanding terminology used in death certificates. However, these findings are in contrast with the study that was performed in Khartoum (El-Nour & Ali, 2007) as the latter study reports that 90% of physicians have a clear and concise knowledge of information mentioned on death certificate forms.

Absence of training can lead to many problems and issues in the process of determining death causes. In addition, lack of adequate information on the formation or incident of death, lack of awareness and experience of filler of death certificate forms, and lack of application of uniform rules and guidelines on determination of death causes have caused errors and mistakes in death causes coding prepared by physicians (responsible for formulating and confirming death certificates) and health information management staffs (accountable for clinical encoders and controllers/assessors). Consequently, inaccurate information and statistics are available about the causes and occurrence of deaths. Therefore, it is expected to be able to address this problem in this country (Iran) by teaching the associated rules and gridlines (Reza, 2005).

The concept underlying cause of death often the source of confusion for process of issuing death certificate by the associated agents. For example, most education and treatment centers as well as hospitals are highly in need of regular studies and enhancement of their death certificate forms. Inspection of personal errors showed that 23.9% of participants had reported death mechanisms correctly. However, this percentage is less than the ones reported by other studies. For instance, in pre-training assessment a study in Canada (Myers, 1998), indicated that 32.9% of physicians had referred to correct death mechanisms. In another study (Smith, 2001), 34% of physicians gave an accurate report of death mechanisms. The aforementioned figures are more than the one reported by the present research.

Specialized assistants while learning (Resident students) probably make all kinds of mistakes especially while determining death underlying cause of death and death multiple causes. Findings of the present research comply with the results obtained by other studies (Alsaleh, Al-Hussein, Almajid, & Alsani, 2008). Beginner practitioners often make errors in completing death certificate forms. Therefore, it is highly recommended to train residents on completion of death certificate forms. Physicians with previous experience of completion of death certificate forms demonstrated a better performance compared to physicians without such an experience. This finding is in line with the finding reported by the study that was carried out in Taiwan (Lu, Shih, Lee, Chou, & Lin, 2001). However, other studies have reported that experience does not solely suffice for improvement of completion of such forms.

In spite of classifications and coding of diseases by education and treatment centers in the past decades, it seems that no serious decision has been made so far by authorities on the application of statistical outputs and information to the decision-making process. Hence, no formal effective measure has been taken to encode death certificates. Only little effort has been made because of educational prejudices and personal interests of health information management staffs. It is proved that the personal judgment of the encoder that results from his/her experiences and knowledge plays an important role in determining the causes of a death and associated information presented in death certificates (Ahmadi & Jahanpour, 2009).

## 5. Conclusion

The quality of the death causes coding performed by the education and treatment centers affiliated with the Ministry of Health and Medical Education is not satisfactorily. Some of the problems can be ascribed to the contents of death certificates and completion of death certificate forms by clinical department faculty staff and residents, while another part can be ascribed to the lack of attention of decision-making authorities to the application of statistical analyses performed by health information management staffs. Due to the aforementioned lack of attention, these experts, as the main drivers of assessment and control of death certificate coding, do not show any interest in this field. Therefore, collection and application of mortality information based on the standards defined by WHO bring about many advantages one of them being the improvement of public health in the society. In order to enhance the awareness and performance of physicians (especially clinical department faculty staff, residents and HIM staffs), it is recommended follow the WHO instructions on mortality rules. The following measures are also recommended to increase the accuracy of diagnoses of death causes and coding:

- Presenting short-term re-training courses to make physicians at all levels familiar with mortality rules and guidelines defined by WHO,
- Presenting a course on coding of diseases and mortality as an obligatory course to all medical students in the long run,
- Holding workshops to make the medical team (especially education and treatment centers managements and hospital administrators) more familiar with mortality rules and guidelines and also to conduct studies aimed at assessing the performance of these agents.
